# Genome-wide mapping of G-quadruplex structures with CUT&Tag

**DOI:** 10.1093/nar/gkab1073

**Published:** 2021-11-18

**Authors:** Jing Lyu, Rui Shao, Philip Yuk Kwong Yung, Simon J Elsässer

**Affiliations:** Science for Life Laboratory, Department of Medical Biochemistry and Biophysics, Karolinska Institutet, Tomtebodavägen 23, 17165 Stockholm, Sweden; Ming Wai Lau Centre for Reparative Medicine, Stockholm node, Karolinska Institutet, Solnavägen 9, 17165 Stockholm, Sweden; Science for Life Laboratory, Department of Medical Biochemistry and Biophysics, Karolinska Institutet, Tomtebodavägen 23, 17165 Stockholm, Sweden; Ming Wai Lau Centre for Reparative Medicine, Stockholm node, Karolinska Institutet, Solnavägen 9, 17165 Stockholm, Sweden; Science for Life Laboratory, Department of Medical Biochemistry and Biophysics, Karolinska Institutet, Tomtebodavägen 23, 17165 Stockholm, Sweden; Ming Wai Lau Centre for Reparative Medicine, Stockholm node, Karolinska Institutet, Solnavägen 9, 17165 Stockholm, Sweden; Science for Life Laboratory, Department of Medical Biochemistry and Biophysics, Karolinska Institutet, Tomtebodavägen 23, 17165 Stockholm, Sweden; Ming Wai Lau Centre for Reparative Medicine, Stockholm node, Karolinska Institutet, Solnavägen 9, 17165 Stockholm, Sweden

## Abstract

Single-stranded genomic DNA can fold into G-quadruplex (G4) structures or form DNA:RNA hybrids (R loops). Recent evidence suggests that such non-canonical DNA structures affect gene expression, DNA methylation, replication fork progression and genome stability. When and how G4 structures form and are resolved remains unclear. Here we report the use of Cleavage Under Targets and Tagmentation (CUT&Tag) for mapping native G4 in mammalian cell lines at high resolution and low background. Mild native conditions used for the procedure retain more G4 structures and provide a higher signal-to-noise ratio than ChIP-based methods. We determine the G4 landscape of mouse embryonic stem cells (ESC), observing widespread G4 formation at active promoters, active and poised enhancers. We discover that the presence of G4 motifs and G4 structures distinguishes active and primed enhancers in mouse ESCs. Upon differentiation to neural progenitor cells (NPC), enhancer G4s are lost. Further, performing R-loop CUT&Tag, we demonstrate the genome-wide co-occurrence of single-stranded DNA, G4s and R loops at promoters and enhancers. We confirm that G4 structures exist independent of ongoing transcription, suggesting an intricate relationship between transcription and non-canonical DNA structures.

## INTRODUCTION

G-quadruplex (G4) structures are composed of three or more stacked G-quartets. Four guanine bases can form a planar G-quartet via Hoogsten hydrogen bonds, and the stacking of G-quartets is stabilized by monovalent cations, typically potassium in a cellular context (1,2). The DNA backbones of the guanines run parallel or antiparallel along the stack, and mixed conformations may exist (3–5). RNA can readily form G4 structures as well, but only a parallel orientation is compatible with the RNA backbone (1). The conformation of G-quadruplex structures is dependent on loop length and loop sequence composition (6): Four GGG-repeats connected by short loops on the same DNA molecule form the canonical intramolecular G4, but G4s have also been shown to fold with longer loops, as few as two or more than three guanine quartets, or with non-G bases breaking up the consecutive G-repeat. Further, GGG-repeats distributed on both strands of a DNA duplex can form inter-strand G4s, and it has been proposed that inter-strand G4s may also form across longer distances via DNA looping (7).

The human genome contains up to half a million predicted G-quadruplex forming sequences (PQS), most of which are found in promoter regions/CpG islands, G-rich tandem repeat regions and telomeres. G4 DNA was first found in telomere regions in ciliates (8,9). Experimental evidence suggests that G4 structures are also enriched in telomeric and sub-telomeric repetitive DNA, ribosomal DNA, promoter regions and interspersed tandem repeats in mammalian cells (10,11). PQS are underrepresented in the coding strand of exons, which indicates that G4 structures in mature mRNA are selected against in evolution (12,13). Nevertheless, RNA G4 structures are thought to regulate mRNA metabolism, and RNA may form hybrid G4s with DNA (1,14).

Initially demonstrated in prokaryotes, G4 structures within promoter regions are implicated in gene regulation (15). G4s have been detected in promoter DNA of oncogenes, such as *c-MYC*, *KRAS* and *c-kit*, and induction of G4s in order to block transcription has been suggested as a strategy to suppress tumorigenesis (16–19). Owing to the unique stacking of the G quartets, many specific small molecule ligands have been developed that specifically intercalate between the planes of G-quartets (20–26): TMPyP4, a commonly used intercalating G4 ligand, has been shown to repress c-MYC and the expression of telomerase reverse transcriptase in mice (27) and leukemia cells (28). Pyridostatin (PDS) has been shown to induce telomere dysfunction, genome-wide DNA-damage and inhibit cancer growth (29,30). Many G4 ligands also intercalate into duplex DNA and/or induce other non-canonical DNA structures, thus raising the question if the observed phenotypes, particularly cytotoxicity, could be unequivocally attributed to G4s (31). Consequently, more selective G4 ligands have been developed in recent years, such as PDC12 (31), CX-3543 (32–35) and CX-5461 (36–38). The latter two have been shown to induce G4s in rDNA, thereby inhibiting RNA polymerase I elongation and rRNA synthesis. Their potent antitumor activity holds great promise for cancer therapy (32,34,36–37). Highlighting the selectivity of such treatment, it has been shown that preexisting endogenous G4 levels in tumors exacerbate sensitivity to G4 ligands (39).

Together, the above-mentioned studies investigating G4 ligand mode of action provide ample evidence that G4s have the capacity to disrupt various DNA-dependent processes. In contrast, the role of endogenous G4s in regulating transcription and replication remains unclear. Promoter G4s correlate with active transcription, but whether and how endogenous G4s fine-tune transcription remains to be determined (39–43). Elegant studies in chicken cells show that individual G4 structures can represent roadblocks to replication (31,44–46), yet such effect has not been confirmed at a genome-wide level.

Methods to precisely map DNA G-quadruplexes in the genome are crucial to study the function and regulation of G4 in physiological and pathological processes. Genome-wide chromatin-immunoprecipitation and sequencing (ChIP-seq)-based mapping methods have been described, using either a G4-specific antibody in an optimized ChIP-Seq protocol (39,41,43,47) or an artificial 6.7 kDa G4 probe (G4P) protein for G4 binding and capture (48). The most common G4 ChIP-Seq protocol entails immunoprecipitation of formaldehyde-fixed and sheared chromatin with the phage-display derived monoclonal BG4 antibody (49), followed by next-generation sequencing (47). Using G4 ChIP-Seq, ∼10 000 DNA G4 structures were mapped on the human genome, enriched in promoters and other regulatory, nucleosome free regions, and G4 formation was correlated with elevated transcriptional activity (43,47). These results highlight the utility of G4 ChIP-Seq to determine genome-wide distribution of G4s. However, it is not clear whether G4 structures are well preserved in the presence of various detergents commonly used in a ChIP-Seq procedure, and whether the required chromatin fragmentation by shearing force may unfold or distort G4 structures. Formaldehyde fixation, intended to preserve G4 structures, may risk epitope-masking, thus reducing G4-specific ChIP enrichment. Therefore, alternative approaches have been sought to capture G4s under more native conditions: the G4P-ChIP method takes advantage of a peptide from the DHX36 helicase with high affinity and specificity for G4 structures, whereas D1 ChIP used a monoclonal antibody-GFP fusion protein for G4 capture (48,50). However, both methods rely on the expression of G4 binders in cells, thus requiring the generation of stable cell lines. Further, it cannot be excluded that the expression of G4 binding proteins affects G4 biology, e.g. by competing with endogenous binding proteins or helicases.

Recently, cleavage under targets and tagmentation (CUT&Tag) has been developed to map chromatin features in permeabilized nuclei using antibody-tethered Tn5 tagmentation (51). In the CUT&Tag procedure, cells or nuclei are permeabilized under mild native conditions. Here, we combined G4 antibody-based detection of genomic G4s with CUT&Tag technology and established a native G4 mapping method termed G4 CUT&Tag. Compared to other G4 mapping methods, G4 CUT&Tag provides superior signal-to-noise ratio and reliability to detect bona-fide G4s. We applied G4 CUT&Tag to mouse embryonic stem cells, observing widespread G4 formation at active genes and enhancers.

## MATERIALS AND METHODS

### Cell culture

HEK293T and HaCaT cells were cultured in DMEM high glucose, GlutaMAX™ Supplement, pyruvate (LifeTechnologies, 10569010), 10% fetal bovine serum (Sigma, F7524) under standard conditions (5% CO_2_, 90% humidity, 37°C). Mouse embryonic stem cells were cultured feeder-free in 0.1% gelatin-coated flasks (Sigma, G1890) under standard conditions (5% CO_2_, 90% humidity, 37°C) in KnockOut DMEM (LifeTechnologies, 10829018), 2 mM alanyl-glutamine (Sigma, G8541), 0.1 mM non-essential amino acids (Sigma, M7145), 15% fetal bovine serum (FBS) (Sigma, F7524), 0.1 mM β-mercaptoethanol (Sigma, M3148), ESGRO Leukemia Inhibitory Factor (LIF) (Millipore, ESG1107), 1 μM PD0325901 (PZ0162-25MG) and 3 μM CHIR99021 (SML1046-25MG). The neural progenitor cells differentiation experiment was performed as described previously (52). For inhibitor treatments, ESC were treated with a final concentration of 100μM DRB (Sigma, D1916-10MG) for 2h or 1μM triptolide (VWR, CAYM11973-1) for 4h.

### BG4 antibody expression and purification

Recombinant FLAG-tagged BG4 antibody and Protein A-Tn5 (pA-Tn5) were purchased from the Protein Science Facility at the Department of Molecular Biochemistry and Biophysics at Karolinska Institutet. Recombinant FLAG-tagged BG4 was produced at the Protein Science Facility in *Escherichia coli* using pSANG10-3F-BG4 (Addgene #55756) (49) as follows: *E. coli* BL21 (DE3) T1R pRARE2 were transformed with pSANG10-3F-BG4 and pre-culture was grown overnight at 30°C in TB, 50 μg/ml Kanamycin, 34 μg/ml Chloramphenicol. 3 l TB, 50 μg/ml Kanamycin, 34 μg/ml Chloramphenicol was inoculated with 45 ml of the overnight culture and grown at 37°C until OD 2, then the culture was shifted to 18°C. At OD 3, IPTG was added to 0.5 mM and expression was carried out overnight at 18°C. Cells were pelleted and resuspended in IMAC lysis buffer by agitation at 4°C (100 mM HEPES, 500 mM NaCl, 10% glycerol, 10 mM imidazole, pH 8.0, 1× complete EDTA-free protease inhibitor cocktail, benzonase) and stored frozen at −80°C. Cells were thawed and disrupted by sonication. The sonicated lysate was centrifuged (20 min at 49 000 g), the supernatant filtered through a 0.45 μm filter and loaded onto a 5 ml HisTrap HP column (GE Healthcare) on a ÄKTA Xpress. The HisTrap column was washed with IMAC wash 1 buffer (20 mM HEPES, 500 mM NaCl, 10% glycerol, 10mM imidazole, pH 7.5), IMAC wash 2 buffer (20 mM HEPES, 500 mM NaCl, 10% glycerol, 50 mM imidazole, pH 7.5) and eluted with IMAC elution buffer (20 mM HEPES, 500 mM NaCl, 10% glycerol, 500 mM imidazole, pH 7.5) directly onto a HiLoad 16/60 Superdex 75 gel filtration column (GE Healthcare) pre-equilibrated with PBS pH 7.4. Gel filtration was run with PBS pH 7.4, and peak fractions were pooled and concentrated. Concentrated (1 mg/ml) FLAG-tagged BG4 was aliquoted and flash-frozen in liquid nitrogen, then stored at −80°C. Three liters TB culture yielded 3.4 mg purified FLAG-tagged BG4.

### Cleavage under targets and tagmentation (CUT&Tag)

CUT&Tag experiments were performed as described previously (51) with minor modifications: 1% BSA (Jackson ImmunoResearch, 001-000-161) was used in the antibody buffer, dig-wash buffer and dig-300 buffer to minimize cell clumping. Briefly, 1 × 10^5^ cells were harvested, washed with wash buffer (20 mM HEPES pH 7.5, 150 mM NaCl, 0.5 mM spermidine), and immobilized to concanavalin A–coated beads (Bangs Laboratories, BP531) with incubation at room temperature for 10 min. The bead-bound cells were incubated in 200 μl of primary antibody buffer (wash buffer with 1% BSA, 2 mM EDTA and 0.05% digitonin for gentle permeabilization of the plasma and nuclear membrane) with 1:100 FLAG-tagged BG4 antibody or S9.6 (Millipore, MABE1095) antibody dilution at 4°C by rotating overnight. The next day, the primary antibody buffer was removed and cells were washed with 800 μl of dig-wash buffer (wash buffer with 1% BSA and 0.05% digitonin) three times. After washing, BG4 antibody-incubated cells were resuspended in 200 ul of dig-wash buffer with 1:100 dilution of mouse anti-FLAG antibody (Sigma, F1804) and incubated at room temperature for 1 h with slow rotation. Cells were washed with 800 μl of dig-wash buffer briefly three times to remove unbound antibodies. S9.6-treated or anti-FLAG treated cells were incubated with 1:100 dilution of rabbit anti-mouse antibody (Sigma, M7023) in 200 μl of dig-wash buffer at room temperature for 1 h with slow rotation.

After a brief wash with dig-wash buffer as above, cells were resuspended in 200 ul of dig-300 buffer (20 mM HEPES pH 7.5, 300 mM NaCl and 0.5 mM spermidine, 1% BSA and 0.01% digitonin) with 1:200 dilution of pA-Tn5 adapter complex and incubated at room temperature for 1 h with slow rotation. pA-Tn5-bound cells were washed with 800 ul of dig-300 buffer three times, followed by tagmentation in 200 ul of tagmentation buffer (dig-300 buffer with 10 mM MgCl_2_) at 37°C for 1 h. After tagmentation, 15 mM EDTA, 500 μg/ml proteinase K and 0.1% SDS were added and further incubated at 63°C for another 1 h to stop tagmentation and digest protein. Genomic DNA was extracted and purified with DNA Clean & Concentrator-5 (Zymo research, D4013). To generate G4 or R-loop libraries, purified genomic DNA was amplified with the universal i5 primer and barcoded i7 primer using NEBNext Ultra II Q5 Master Mix (NEB, M0544). The library PCR products were cleaned up with Agencourt AMPure XP beads (Beckman Coulter, A63881) and sequenced on an Illumina Nextseq 500 instrument.

For Mung Bean nuclease (NEB, M0250L) treatment, concanavalin A–coated beads-bound cells were incubated at 30°C for 30 min with 100 U or 150 U Mung Bean nuclease in 200 ul of dig-wash buffer prior to G4 or R-loop primary antibody incubation.

For H3K4me3 or H3K27me3, CUT&Tag was performed as above using H3K4me3 (Millipore, 04-745) or H3K27me3 (Millipore, 07-449) primary antibody and guinea pig anti-rabbit (Antibodies-Online, ABIN101961) as secondary antibody.

### *Drosophila* S2 spike-in control

A spike-in was used where indicated to provide an external scaling factor for quantitative comparison across conditions. Briefly, S2 (*Drosophila melanogaste*r) cells were grown at 28°C in Schneider's *Drosophila* Medium (Thermo Scientific, 21720024) with 10% fetal bovine serum (Sigma, F7524) and harvested by centrifugation. G4 CUT&Tag was performed on S2 cells in parallel with the human/mouse samples. After tagmentation and genomic DNA purification, the tagmented DNA was quantified using Qubit assay. The tagmented samples were normalized to the same DNA concentration, and a constant amount (5% of sample) of tagmented *Drosophila* DNA (‘spike-in’) was added to each sample, at a ratio of 1:20. Afterwards, library PCR was performed as described above. An alternative method, mixing 5% S2 cells into the harvested cells before CUT&Tag procedure was also evaluated but showed larger variation due to the error attached to cell counting.

### Mapping pipeline

G4 and R-loop CUT&Tag datasets were processed as follows: reads were aligned with bowtie2 (v.2.3.5.1) (53), trimming mosaic adaptor sequences using the -5 19 parameter, and stored as BAM file using samtools (v1.10) (54). BAM files were deduplicated with picard (v2.23.4) MarkDuplicates. Blacklisted regions were removed from the BAM file with bedtools (v2.29.2) intersect (55) using ENCODE blacklist bed files for mm9 or hg19. Normalized (RPGC, 1x Genome Coverage) coverage tracks were generated using deepTools (v3.3.2) bamCoverage (56) using parameters -binSize 5 –normalizeUsing RPGC and the respective genome size. Peaks were called with MACS2 (v2.2.6) (57). High confidence peaks sets were concordant peaks between three replicates (G4s) or two replicates (R-loops) generated with bedtools (v2.29.2) intersect command and plotted with R package ‘VennDiagram’ (58). Significance (Monte-Carlo FDR) of overlap between peaksets was tested against randomized intervals using GSuite HyperBrowser (v2.1.3) (59).

### Annotation of G4 CUT&Tag peaks

A published 15-state ChromHMM model generated with seven histone modifications and RNA Polymerase II (RNAP2) profiles was used to annotate G4 CUT&Tag peaks (60). Peak intervals were overlapped with the ChromHMM annotation with bedtools (v2.29.2) intersect command, and the fraction of G4 peaks overlapping with each state was calculated.

### Promoter and enhancers definitions

Transcription start sites were extracted from RefSeq genes and deduplicated to define the promoter set. Published active, primed and poised enhancer sets were used (61). In this study, combinations of ChIP-Seq peaks were used to define active (p300 + H3K27ac), primed (H3K4me1) and poised (p300 + H3K27me3) classes. For heatmaps, gene promoter and enhancer regions were defined as ±3 kb from the transcription start site or center of enhancer, respectively.

### G4 motif analysis

Top 1000 MACS2-called G4 peaks were extracted based on the score. The DNA sequence underlying the peaks intervals were extracted with the bedtools (v2.29.2) getfasta command and G4 sequence motifs were searched with MEME suite online tool (62).

### Quality metrics

FriP was calculated with Subread (v2.0.3) featureCounts (63). Fingerplots were generated with deepTools (v3.3.2) plotFingerprint (56).

### Telomeric sequence content

To determine the fraction of telomeric reads in the CUT&Tag library, FASTQ files for mapped against a pseudogenome of repetitive sequences using bowtie2 (v.2.3.5.1) and the number of reads mapping to the telomeric repeat sequence (20xTTAGGG) was extracted with samtools (v1.10) idxstats command.

### Analysis and visualization of data

Analyses were generated from normalized bigwig files. Profile plots and heatmaps were generated with SeqPlots (v1.10.6) (64). Scatterplots and ChromHMM heatmaps were generated using wigglescout (https://github.com/cnluzon/wigglescout/), an R library for bigWig genomics data visualization.

### G-quadruplex motif search

Genomic sequences of mm9 and hg19 were subjected to two classes of G4 pattern matching, inter-strand motifs (65) and intra-strand motifs (23). For inter-strand motifs, the canonical G4 motif G_3+_L_1-7_ was expanded to the opposite strand in 8 combinations. Let A = G_3+_ and B = C_3+_, the 8 patterns are AAAA, AAAB, AABA, AABB, ABAA, ABAB, ABBA and ABBB. The canonical intra-strand G4 pattern was the same as AAAA; the extra intra-strand patterns were extended canonical PQS (Putative G-Quadruplex Sequences) G_3+_L_1–12_, and two-tetrads PQS G_2_L_1–12_. Regular expression was applied, for example two-tetrads PQS was ‘[gG]{2}\\w{1,12}{3,}[gG]{2}’. Motif genome coverage was generated with R package ‘rtracklayer 1.46.0’.

### Reference datasets

Public data used in this study were downloaded from GEO: GSM2035780 (HaCaT G4 ChIP-seq), GSM2035782 (HaCaT G4 ChIP-seq, input), GSM3907020 (HEK293T G4P-ChIP), GSM3907021 (HEK293T G4P-ChIP, input), GSM1917298 (ESC Ring1b ChIP-seq), SRR10349547 (ESC KAS-seq), GSM1173376 (ESC S2 PolII ChIP-seq), GSM4205678 (ESC H3K27ac ChIP-seq), GSM4303796 (ESC H3K4me1 ChIP-seq), GSM4661960 (ESC ATAC-seq), GSM1127953 (ESC Bisulfite-seq) (66), GSM2582392 (ESC H3K9me3 ChIP-seq), GSM789450 (NPC H3K4me1 ChIP-seq), GSM1516096 (NPC H3K27ac ChIP-seq), GSM2417142 (ESC Oct4 ChIP-seq), GSM2417143 (ESC Sox2 ChIP-seq), GSM2417144 (ESC Klf4 ChIP-seq). From the downloaded FASTQ files, reads were aligned with bowtie2 (v.2.3.5.1) (53) and samtools (v1.10) (54). BAM files were deduplicated with picard (v2.23.4) MarkDuplicates. Blacklisted regions were removed from the BAM files with bedtools (v2.29.2) intersect (55) using ENCODE blacklist bed files for mm9 or hg19. Normalized (RPGC, 1x Genome Coverage) coverage tracks were generated using deepTools (v3.3.2) bamCoverage (56) using parameters -binSize 5 –normalizeUsing RPGC. CpG density track was built by generating a bed file for all CG dinucleotide sequences in the mm9 genome.

## RESULTS

### Systematic comparison of G4 CUT&Tag with other G4 mapping methods

**Figure 1.**
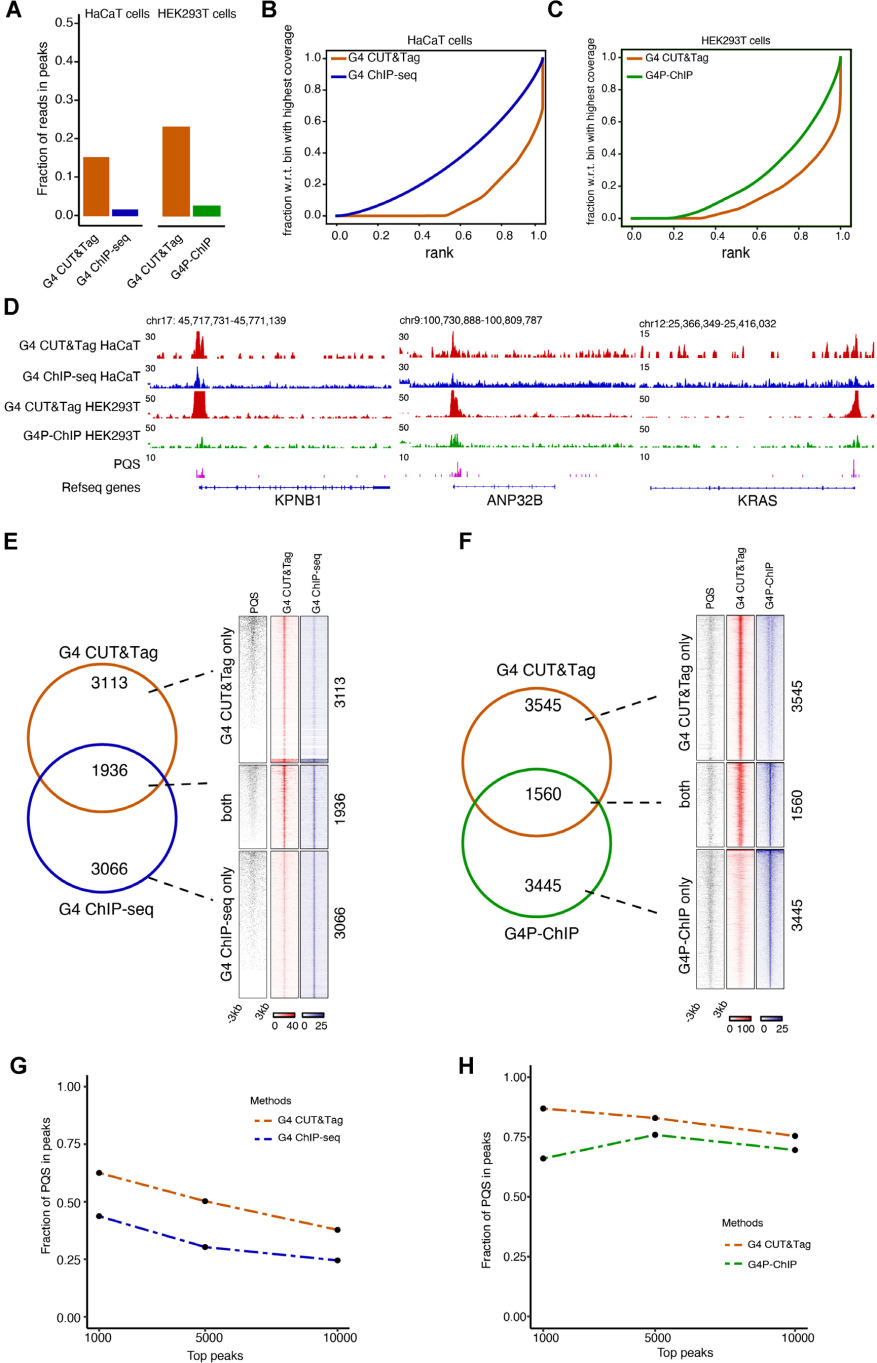
Comparison of G4 CUT&Tag to G4 ChIP-seq and G4P-ChIP. (**A**) Comparison of fraction of reads in G4 peaks between G4 CUT&Tag library generated in this study and from public G4 ChIP-seq dataset in HaCaT cells, or public G4P-ChIP dataset in HEK293T cells (43,48). (**B**) Fingerprint (cumulative read count sum by ranked bins) plot of G4 CUT&Tag and G4 ChIP-seq in HaCaT cells (43). (**C**) Fingerprint (cumulative read count sum by ranked bins) plot of G4 CUT&Tag and G4P-ChIP in HEK293T cells (48). (**D**) Genome browser view of G4 signals at example loci. The RPGC-normalized (1× Genome Coverage) tracks of G4 CUT&Tag and G4 ChIP-seq in HaCaT cells, or G4 CUT&Tag and G4P-ChIP in HEK293T cells are shown on the same y-axis scale. (**E)** Overlap of top 5000 G4 CUT&Tag and G4 ChIP-seq peaks. Total PQS, G4 CUT&Tag and G4 ChIP-seq density heatmaps for peaks classified as G4 CUT&Tag only (*n* = 3113), both G4 CUT&Tag and G4 ChIP-seq (*n* = 1936) or G4 ChIP-seq only (*n* = 3066). (**F**) Overlap of top 5000 G4 CUT&Tag and G4P-ChIP peaks. Total PQS, G4 CUT&Tag and G4P-ChIP density heatmaps for peaks classified as G4 CUT&Tag only (*n* = 3545), both G4 CUT&Tag and G4P-ChIP (*n* = 1560) or G4P-ChIP only (*n* = 3445). (**G**) Comparison of fraction of PQS (canonical or non-canonical) in top 10 000, top 5000, and top 1000 G4 peaks between G4 CUT&Tag and G4 ChIP-seq in HaCaT cells. (**H**) Comparison of fraction of PQS (canonical or non-canonical) in top 10 000, top 5000 and top 1000 G4 peaks between G4 CUT&Tag and G4P-ChIP in HEK293T cells.

We established G4 CUT&Tag starting with version 2 of CUT&Tag (51) with minor modifications. We expressed and purified recombinant FLAG-tagged BG4 scFv anti-G-quadruplex antibody, originally derived from a phage display library (49). Permeabilized cells were incubated sequentially with FLAG-tagged BG4 antibody, mouse anti-FLAG antibody, rabbit anti-mouse IgG antibody and finally recombinant Protein A-Tn5 (pA-Tn5) fusion protein to achieve tagmentation of G4-containing chromatin fragments. To systematically compare the G4 CUT&Tag with other G4 mapping methods, we performed G4 CUT&Tag in HEK293T and HaCaT cells for which prior genome-wide datasets had been generated. In parallel, we reprocessed the raw data for G4 ChIP-seq (43), G4P-ChIP (48) through the same bioinformatic pipeline as our G4 CUT&Tag data. G4 CUT&Tag demonstrated improved raw data quality compared with both existing methods, yielding a tenfold higher fraction of reads under peaks (FriP) than G4 ChIP-seq and sixfold higher FriP than G4P-ChIP (Figure 1A). Fingerprint plots also demonstrated a higher signal-to-noise ratio from G4 CUT&Tag (Figure 1B, C). Examples show the large pile-up of G4 CUT&Tag on promoters with predicted G4 sequences (Figure 1D). We further evaluate the overlap of peaks identified by G4 CUT&Tag with peaks identified by G4 ChIP-seq or G4P-ChIP. To allow an unbiased comparison, the top 10 000, top 5000 and top 1000 scoring peaks identified by each method were extracted. Between G4 CUT&Tag and G4 ChIP-seq, 33% of top 10 000 peaks, 38% of top 5000 peaks and 30% of top 1000 peaks were identified by both methods, respectively (Figure 1E; Supplementary Figure S1). Between G4 CUT&Tag and G4P-ChIP, 45.83% of top 10 000 peaks, 32.76% of top 5000 peaks and 11.7% of top 1000 peaks are identified by both methods, respectively (Figure 1F; Supplementary Figure S1A, B). While the relatively small overlap would suggest a substantially different distribution of peaks, assessing the read density under uniquely called peaks confirmed that they showed co-enrichment in the respective other condition even if they do not meet the threshold for peak calling (Figure 1E, F; Supplementary Figure S1C, D). Nevertheless, differences existed between the quality of the called peaks: For those top peaks unique to each method, the G4 CUT&Tag protocol generated a higher percentage of peaks that match predicted G4 sequences (PQS, including inter-strand and intra-strand G4s) (Figure 1G, H). This further demonstrates that G4 CUT&Tag protocol allows mapping G4 structures with higher confidence than immunoprecipitation-based methods. Our G4 CUT&Tag data was generated from 1 × 10^5^ cells, while 1 × 10^7^ and 1 × 10^8^ cells were typically used for G4 ChIP-seq and G4P-ChIP. In summary, G4 CUT&Tag enabled a significant improvement in signal-to-noise ratio with much lower input requirements, as compared to G4 ChIP-seq and G4P-ChIP.

### Characterization of G4s in mouse ESC

Next, we performed G4 CUT&Tag in mouse embryonic stem cells (ESC). ESCs are pluripotent stem cells featuring an open and dynamic chromatin structure that maintains an uncommitted state poised for differentiation (67). G4 structures have been proposed to play a regulatory role in development (68), but the G4 landscape of ESCs has not been elucidated to date.

**Figure 2.**
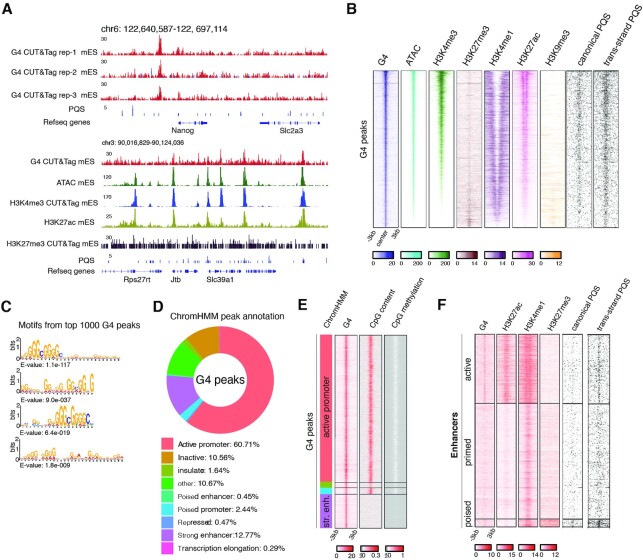
G4 landscape in mouse embryonic stem cells. (**A**) Genome browser view showing triplicate G4 CUT&Tag experiment in ESCs at *Nanog* locus, and comparison of G4 CUT&Tag, H3K4me3 and H3K27me3 CUT&Tag from the same cells, as well as published ATAC-seq (71) and H3K27ac ChIP-Seq (87). (**B**) Density heatmaps of the same tracks at high-confidence (shared between three replicates) G4 peaks in ESCs. Prediction of canonical and non-canonical (trans-strand) PQS are shown. (**C**) Motif discovery using MEME suite from top 1000 G4 peaks shows typical canonical and non-canonical G4 patterns. (**D**) Annotation of high-confidence G4 peaks with different functional genomic features as defined by ChromHMM (70). (**E**) Density heatmaps of G4 CUT&Tag, CpG content and methylation and G4 peaks grouped by ChromHMM annotation as in (D). (**F**) Density heatmaps of G4 CUT&Tag, H3K27ac, H3K4me1, H3K27me3, canonical and non-canonical PQS at active enhancer (active), primed enhancer (primed) and poised enhancer (poised) regions (61).

We verified G4 CUT&Tag in ESCs to be robust and reproducible across triplicate experiments (Figure 2A, Supplementary Figure S2A,B). 9186 high-confidence G4 CUT&Tag peaks were identified by intersecting peaks called from the three replicates, showing good overlaps with canonical and non-canonical (trans-strand) PQS (Figure 2B). To further validate the specificity of G4 CUT&Tag for G4 motifs, we interrogated the top 1000, top 500 and top 200 G4 peaks for recurring motifs using MEME suite and confirmed a high prevalence of G-rich sequences among the top peaks (Figure 2C). G4 CUT&Tag peaks predominantly associated with active promoters (61%) and enhancers (13%), and coincided with open chromatin, H3K4me3, H3K27ac and H3K4me1 (Figure 2B, D). Importantly, a CUT&Tag control experiment with a H3K27me3 antibody showed enrichment at bivalent but not active TSS, demonstrating that the strong G4 CUT&Tag enrichment over active promoters and enhancers does not reflect a purely technical bias for open chromatin. A small proportion (3%) of G4s also coincided with repressed or bivalent promoters marked by H3K27me3 (Figure 2B, C, Supplementary Figure S2C, D). G4 CUT&Tag profiles on gene-coding regions showed a strong peak around the transcription start sites (TSS), while largely absent in the gene body despite the presence of PQS (Supplementary Figure S2B). During RNA Polymerase passage, Spt6, FACT and other histone chaperones maintain dense nucleosome occupancy in gene bodies (69), thus likely disfavoring formation of G4. Promoters of non-expressed genes with neither H3K4me3 nor H3K27me3 showed the lowest G4 signal (Supplementary Figure S2D). Consistent with observations in human cells (41), G4s were positively correlated with CpG density, and reversely correlated with CpG methylation (Supplementary Figure S2E-H).

Enhancers are much less G-rich than gene promoters, however they were still amongst the most abundant G4-enriched regions (Figure 2E). To further investigate this conundrum, we assessed G4s across a reference list of active, primed or poised enhancers (61): G4s occured at active and poised, but not primed enhancers (Figure 2F). Notably, enhancers lacked canonical G4-motifs. Instead, non-canonical G4 motifs constitute G4 peaks at both active and poised enhancers (Figure 2F), and G4 motifs were essentially absent from primed enhancers. Thus, surprisingly, the presence/absence of G4 motifs is a differentiating feature of active and primed enhancers in ESC (Figure 2F).

We wondered how the G4 landscape would change upon exit of pluripotency. ESC can be differentiated into a wide range of lineage-specific stem cells through defined *in vitro* culture protocols. We derived neural progenitor cells (NPCs) from ESC and sought to compare their G4 profiles. Comparing cell types with substantially different morphology, cell cycle and nuclear organization, we wanted to establish a spike-in normalization strategy to capture quantitative difference with CUT&Tag. We considered mixing *Drosophila* S2 cells with ESC in a defined ratio before the CUT&Tag procedure, but pilot experiment hinted that imprecise cell counting generated unwanted spike-in variability. Hence, we performed G4 CUT&Tag on *Drosophila* S2 cells in parallel to ESC and NPCs, and spiked a precisely quantified amount of tagmented *Drosophila* S2 genomic DNA into each tagmented ESC and NPC sample. Genome tracks can then be scaled quantitatively relative to the constant *Drosophila* S2 content present in each sample. We termed this method quantitative or qCUT&Tag.

**Figure 3.**
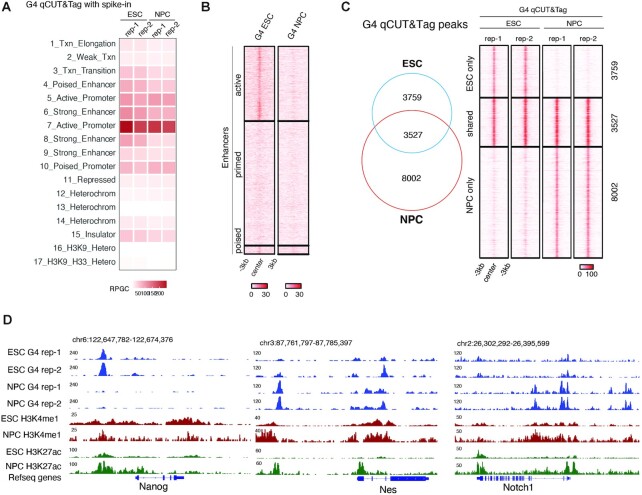
Quantitative comparison of G4 landscapes with G4 qCUT&Tag. (**A**) G4 qCUT&Tag signal across 17 chromatin states in mouse ESC and NPC. (**B**) Density heatmaps of G4 qCUT&Tag in ESC and NPC at active enhancer (active), primed enhancer (primed) and poised enhancer (poised) regions as defined in ESC (61). (**C**) Venn diagram and density heatmaps showing intersection of G4 qCUT&Tag peaks from ESC and NPC. (**D**) Genome browser view showing G4 qCUT&Tag, H3K4me1 and H3K27ac tracks for *Nanog* and neuronal lineage-specific genes *Nes* and *Notch1*.

An analysis across ChromHMM-annotated functional regions (70,71) showed that the genome-wide abundance of G4s largely remained unchanged upon differentiation of ESC to NPC (Figure 3A). G4 levels at poised promoters, active genes, and insulators remained stable whereas strong enhancers lost G4 qCUT&Tag signal (Figure 3A). Intriguingly, active enhancers in ESC, many of which maintained the active enhancer marks H3K4me1 and H3K27ac (Supplementary Figure S2I), collectively lost G4s (Figure 3B, D). Primed enhancers in ESC did not gain G4s in NPC despite many of them acquired H3K27ac (Supplementary Figure S2I). This is in line with our observation that primed enhancers lack PQS (Figure 2F). Overall, ESC and NPC shared a fraction of constitutive G4 qCUT&Tag peaks whereas each cell type had a larger set of unique G4s (Figure 3C), associated with gene expression changes. For example, pluripotency factor Nanog features an ESC-specific G4 at a proximal enhancer, whereas neural lineage-specific genes Nes and Notch1 gain G4 signal (Figure 3D).

In summary, applying G4 qCUT&Tag to mouse ESC and NPCs demonstrated the utility of the method for qualitative and quantitative comparison of genomic G4 landscapes. We discovered that G4s are a common feature of active but not primed enhancers in ESC.

### G4 CUT&Tag signals are sensitive to single-strand specific nuclease digestion

**Figure 4.**
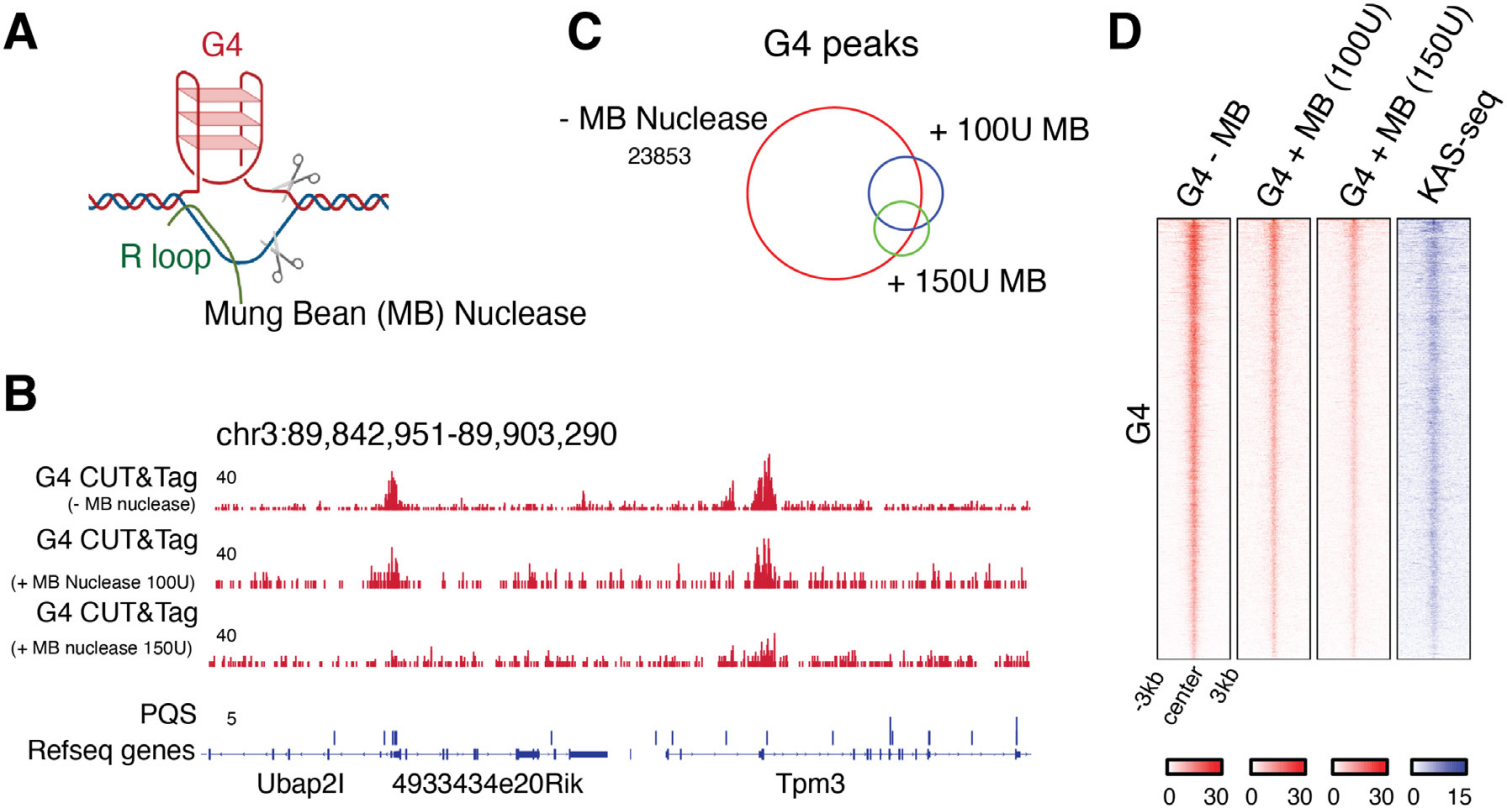
Single-strand specific endonuclease treatment. (**A**) Mung Bean (MB) nuclease treatment inhibits G4 CUT&Tag amplification. (**B**) Genome browser view showing G4 CUT&Tag signals in ESCs with or without Mung Bean nuclease pretreatment. (**C**) Venn diagram showing overlap of G4 CUT&Tag peaks from native, 100U of MB treatment and 150U of MB treatment conditions. (**D**) Density heatmaps of native G4 CUT&Tag, 100U MB-treated G4 CUT&Tag,150U MB-treated G4 CUT&Tag, and single-stranded DNA from KAS-seq (72) at high-confidence G4 peak regions.

G4s formed by one DNA strand leave the C-rich opposite strand single-stranded (Figure 4A). Indeed, methods to map single-stranded DNA (ssDNA) reported widespread ssDNA formation at active TSS (72,73). A recent study using kethoxal-assisted single-stranded DNA sequencing (KAS-seq) showed that ssDNA regions were maintained even after inhibition of transcriptional elongation or depletion of RNA Polymerase II, suggesting that the generation and stabilization of ssDNA does not require transcription (72). Indeed, replotting the ESC KAS-seq data confirmed ssDNA existence at G4 peaks (Figure 4D), and we found a positive correlation between KAS-seq and G4s across TSS (Supplementary Figure S4A). We wondered if we can corroborate the co-existence of G4 and ssDNA, and thus indirectly validate G4 CUT&Tag signals, by applying a single-strand specific endonuclease to permeabilized cells, before subjecting to G4 CUT&Tag. Mung Bean nuclease is a single-strand DNA/RNA-specific endonuclease (74). Digestion of ssDNA by Mung Bean nuclease should disrupt G4 structures and/or inhibit PCR amplification of the G4-associated, tagmented DNA (Figure 4A). ESC were pretreated with 100 units and 150 units of Mung Bean nuclease. G4 CUT&Tag signals decreased at TSS regions after Mung Bean nuclease nuclease treatment, and analysis of high-confidence G4 peaks confirmed that Mung Bean nuclease treatment led to substantial reduction of G4 signals across all G4 CUT&Tag peaks (Figure 4B–D). After 100 units and 150 units of Mung Bean nuclease treatment, G4 CUT&Tag peak numbers decreased by 78.6% and 88.3% respectively (Figure 4C).

### G4s are paired with R loops genome-wide in ESC

**Figure 5.**
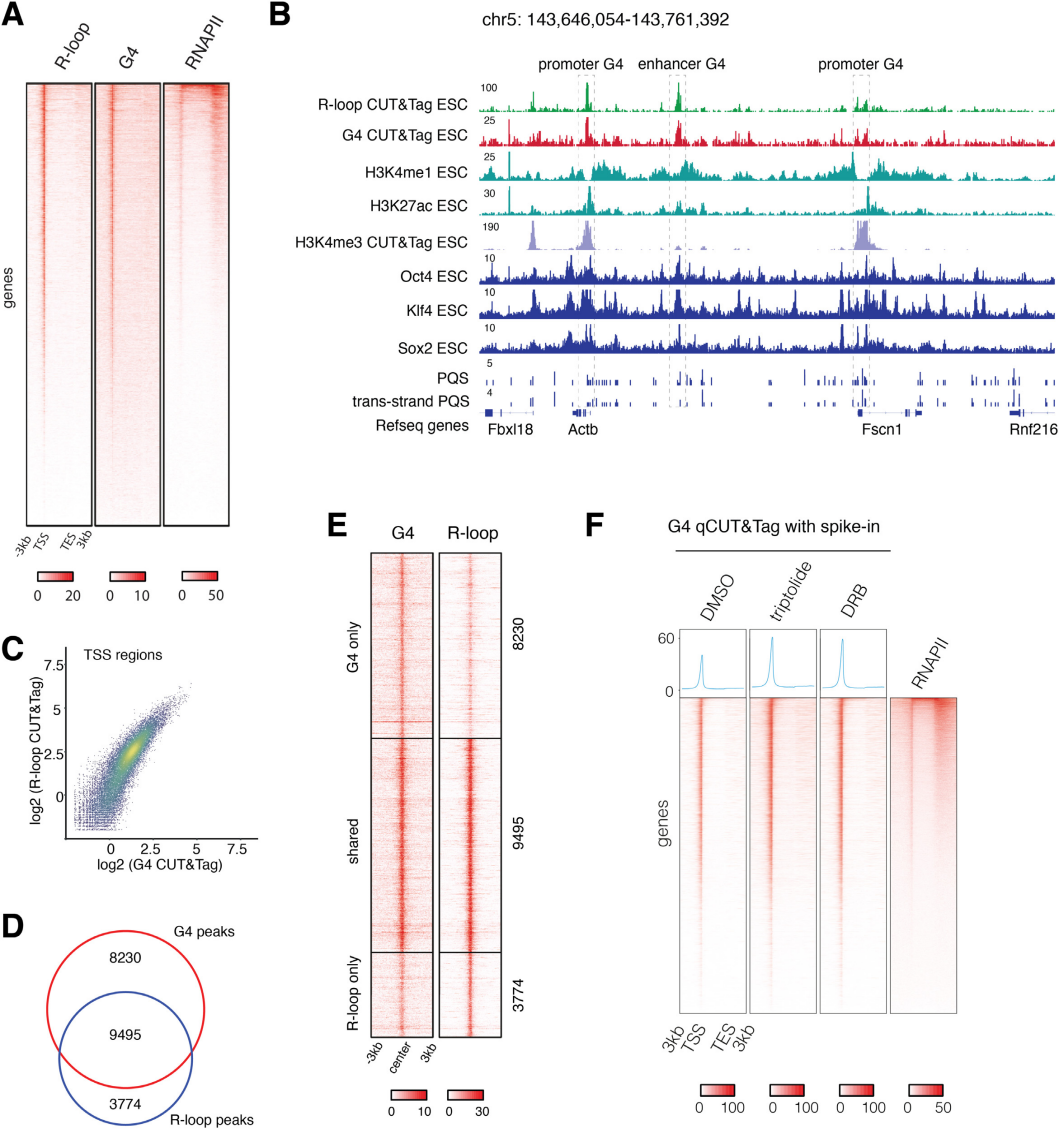
Genome-wide coincidence of R-loops and G4s. (**A**) Density heatmaps of R-loop CUT&Tag, G4 CUT&Tag, RNA Pol II ChIP-Seq (88) at genes. (**B**) Genome browser view showing coincidence of R-loop and G4 at promoter and enhancer regions. (**C**) Scatterplot showing relationship of G4 CUT&Tag and R-loop CUT&Tag signal at TSS. Pearson correlation coefficient was calculated. (**D**) Intersection of G4 CUT&Tag and R-loop CUT&Tag peaks. (**E**) G4 and R-loop CUT&Tag density heatmaps for peaks classified as G4 only (*n* = 8230), both G4 and R-loop (*n* = 9495), or R-loop only (*n* = 3774). (**F**) Density heatmaps of G4 qCUT&Tag in DMSO, triptolide or DRB treated ESC, and RNA Pol II ChIP-Seq (88) at genes.

DNA-RNA hybrids (R loops) formed by the opposing C-rich strand with nascent transcripts are thought to promote and stabilize G4 structures, and vice versa (26). Various R-loop mapping methods have been described to date, generating substantially different profiles: R-loops are most abundant in AT-rich regions in S1-DRIP-seq method (75), but most of the other DRIP-seq methods established a prevalence of R-loops at CG-rich regions (76–78). We thus wondered if R-loop can also be detected with the CUT&Tag protocol using an R-loop specific S9.6 monoclonal antibody (79). In line with a recent report validating the utility of S9.6 CUT&Tag for mapping R loops (80), we achieved a high signal-to-noise ratio and determined R loops genome-wide in ESCs (Figure 5A). Like G4, R-loop CUT&Tag peaks were also sensitive to Mung Bean nuclease treatment (Supplementary Figure S3C). R-loops mapped with S9.6 CUT&Tag were found at active promoters which also exhibited high RNA Pol II occupancy (Figure 5A). High-confidence R-loop CUT&Tag peaks, generated from the intersection of two replicates, predominantly overlapped with active promoters (34%) and enhancers (20%) (Figure 5B, Supplementary Figure S4A-D). Comparing G4 and R-loop CUT&Tag signals revealed that R-loop showed positive correlation (*r* = 0.78) with G4 at TSS regions (Figure 5C), and 71% R-loop peaks overlapped with G4 peaks (*P* = 0.004) (Figure 5D). Plotting G4 and R-loop signals across shared, G4-only and R-loop only peaks supported their large overlap (Figure 5E). These results collectively demonstrate that G4 and R-loop show high degree of co-occurrence. Interestingly, while a recent in vitro study suggested that R-loops and G4s form as a consequence of transcription and contribute mutually to their stability (81), R-ChIP and G4 ChIP studies have found that promoter R-loops and G4s do not require ongoing transcription for their maintenance (42,78). We performed G4 qCUT&Tag in mouse ESC treated with DRB, triptolide or a DMSO control. We observed an slight increase in G4 qCUT&Tag signal over promoters and enhancers upon inhibition of transcription using either DRB or triptolide (Figure 5F, Supplementary Figure S4E, F), corroborating prior reports that ssDNA, R-loops, G4s are stable even in the absence of ongoing transcription (42,72,78).

## DISCUSSION

Genome-wide mapping methods are critical for understanding G4 biology. Here, we demonstrate that CUT&Tag provides a reliable platform for detecting G4s across human and mouse genomes. Compared to published methods, G4 CUT&Tag shows greater sensitivity and a higher fraction of bona-fide G4 peaks with G4 motifs (Figure 1G, H). Validation with single-stranded nuclease pre-treatment demonstrates that the observed G4 CUT&Tag signals arise from regions where the DNA duplex is melted (Figure 4A). During revisions of our manuscript, another study also reported development of G4 CUT&Tag in human cells, providing complementary validation for the robustness of the method (82).

Limitations of the CUT&Tag procedure exist and are also relevant for mapping G4 structures: currently, the protocol crucially relies on native cells and has not been shown to work with fixed tissues, as has been demonstrated for G4 ChIP-Seq (39). While we demonstrate an excellent signal-to-noise ratio measured against other available methodologies, the background of a G4 mapping method should theoretically be zero for regions without any PQS. Our Mung Bean nuclease experiment shows that in the absence of G4 structures, the BG4 antibody still produces a background of random genomic tagmentation events (remaining reads shown in bottom track of Figure 4B). In contrast, omitting BG4 antibody in the CUT&Tag procedure leads to a strong reduction in global tagmentation efficiency, producing only low-complexity libraries (Supplementary Figure S5).

The lack of an input control for the CUT&Tag signal makes it difficult to interpret signals over repetitive sequences. G4s have been suggested to form at interspersed G-rich tandem repeat regions and telomeres *in vivo*, thus quantification of G4 signals at such regions would be desirable as part of a genome-wide study. Telomeric sequences were detected in G4 CUT&Tag libraries at 0.2% abundance and a reduction of telomeric reads with Mung Bean nuclease suggested that G4 CUT&Tag was indeed detecting telomeric G4 (Supplementary Figure S6A). However, we noted that the proportion of telomeric sequences across CUT&Tag replicates was variable across replicates (Supplementary Figure S6B, C).

Despite the abundance of G4-forming motifs present across human and mouse genomes, we find that formation of stable G4s requires open chromatin, as found at active promoters and enhancers. Interestingly, while canonical G4 motifs are more prevalent at promoters, we find that enhancers more often feature inter-strand G4 folding (Figure 2E). A model supported by in silico and in vitro data suggests that G4s can fold in trans at chromatin loop anchors, e.g. with contribution of two GGG repeats by the promoter and two by the enhancer (83,84). The existence of such trans-loop or ‘kissing’ loop G4s in cells is difficult to test experimentally but it is intriguing to speculate that such G4s could stabilize promoter-enhancer interactions through a direct tethering of the DNA (83,84). Interestingly, we find that only embryonic active enhancers feature non-canonical G4 motifs, whereas primed enhancers (carrying H3K4me1 but not H3K27ac) generally lack G4 motifs. The underlying reason for such sequence-encoded property is yet to be elucidated but we note that the pluripotency-associated transcription factor KLF4 recognizes GpG dinucleotides within a G-rich context (85,86). Hence, G4s may participate in a unique enhancer architecture in pluripotent embryonic stem cells.

In summary, we have shown here that CUT&Tag provides a reliable and simple approach for genome-wide mapping of G4 structures and R loops, and we envision that the method will be generally useful for mapping of non-canonical DNA structures for which specific antibodies or specific binding modules are available.

## DATA AVAILABILITY

Associated code is available at https://github.com/elsasserlab/G4.

CUT&Tag data generated for this manuscript has been deposited at the Gene Expression Omnibus under GSE173103.

## Supplementary Material

gkab1073_Supplemental_File
