# Supplementary material for: Genome-wide mapping of G-quadruplex structures with CUT&Tag
Source: Nucleic Acids Res. 2021 Nov 18;50(3):e13. doi: 10.1093/nar/gkab1073 (PMC8860588; doi:10.1093/nar/gkab1073)
Supplement: gkab1073_Supplemental_File [file gkab1073_supplemental_file.pdf]

Genome-wide mapping of G-quadruplex structures with CUT&Tag

Jing Lyu[1,2], Rui Shao[1,2], Philip Yuk Kwong Yung[1,2], Simon J Elsässer[1,2]

Affiliations

[1] Science for Life Laboratory, Department of Medical Biochemistry and Biophysics, Karolinska Institutet, Tomtebodavägen 23, 17165 Stockholm, Sweden

[2] Ming Wai Lau Centre for Reparative Medicine, Stockholm node, Karolinska Institutet, Solnavägen 9, 17165 Stockholm, Sweden

Corresponding Author

Simon J Elsässer, simon.elsasser@scilifelab.se

SUPPLEMENTARY MATERIAL

Supplementary Figure 1

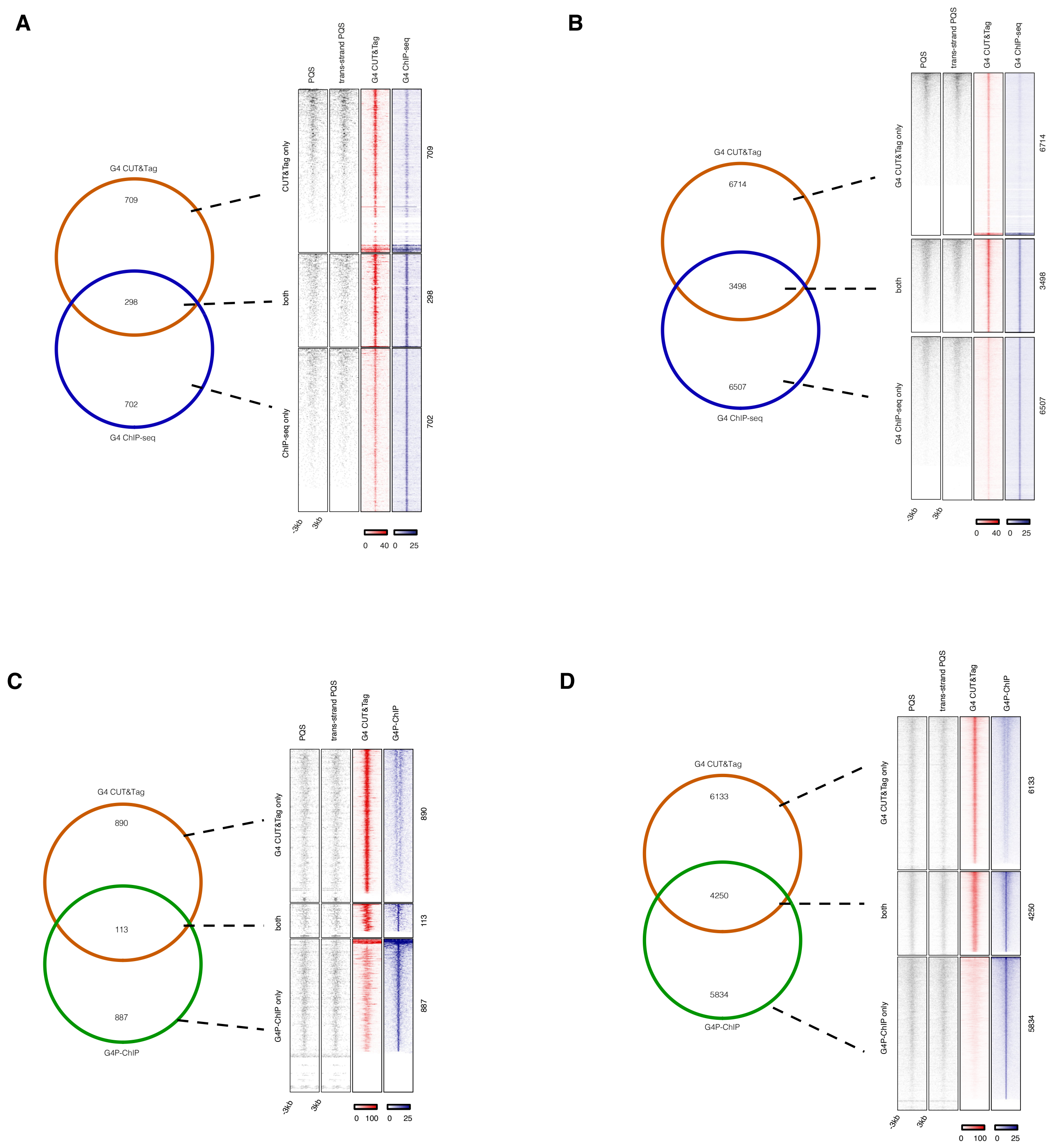

Supplementary Figure 1: Comparison of G4 CUT&Tag to other G4 mapping methods, G4 ChIP-seq and G4P-ChIP. (**A**) Overlap of top 1000 G4 CUT&Tag and G4 ChIP-seq peaks. Total PQS, trans-strand PQS, G4 CUT&Tag and G4 ChIP-seq density heatmaps for peaks classified as G4 CUT&Tag only (n = 709), both G4 CUT&Tag and G4 ChIP-seq (n = 298) or G4 ChIP-seq only (n = 702). (**B**) Overlap of top 10,000 G4 CUT&Tag and G4 ChIP-seq peaks. Total PQS, trans-strand PQS, G4 CUT&Tag and G4 ChIP-seq density heatmaps for peaks classified as G4 CUT&Tag only (n = 6714), both G4 CUT&Tag and G4 ChIP-seq (n = 3498) or G4 ChIP-seq only (n = 6507). (**C**) Overlap of top 1000 G4 CUT&Tag and G4P-ChIP peaks. Total PQS, trans-strand PQS, G4 CUT&Tag and G4 ChIP-seq density heatmaps for peaks classified as G4 CUT&Tag only (n = 890), both G4 CUT&Tag and G4 ChIP-seq (n = 113) or G4 ChIP-seq only (n = 887). (**D**) Overlap of top 10,000 G4 CUT&Tag and G4P-ChIP peaks. Total PQS, trans-strand PQS, G4 CUT&Tag and G4 ChIP-seq density heatmaps for peaks classified as G4 CUT&Tag only (n = 6133), both G4 CUT&Tag and G4 ChIP-seq (n = 4250) or G4 ChIP-seq only (n = 5834).

Supplementary Figure 2

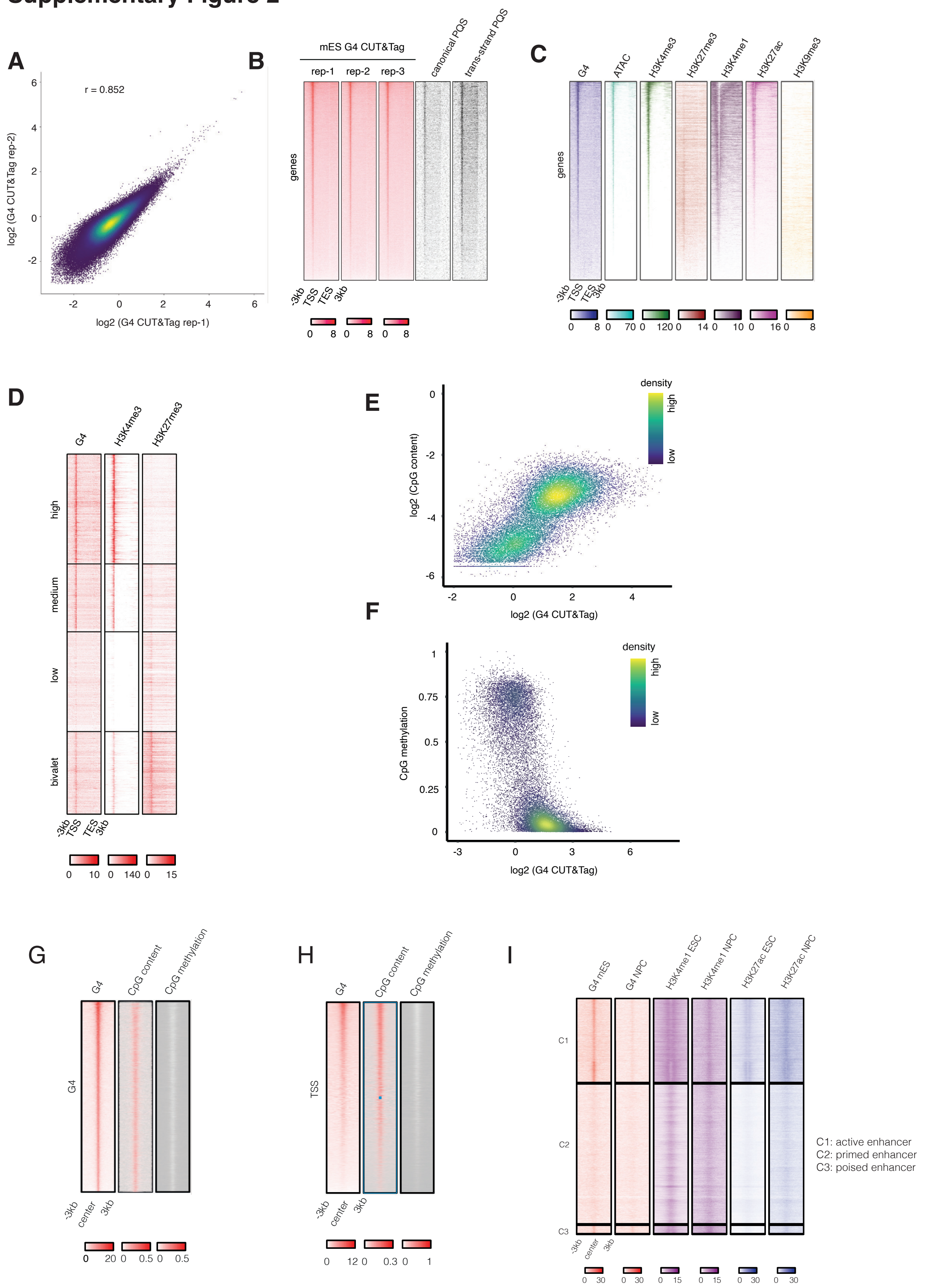

Supplementary Figure 2: **G4 landscape in mouse embryonic stem cells.** (**A**) Scatterplot of 10kb windows genome-wide showing the reproducibility of G4 CUT&Tag signal between two ESC replicates. Pearson correlation was calculated. (**B**) Density heatmaps showing the reproducibility of G4 CUT&Tag at gene-coding regions, and overlap with canonical and non-canonical (trans-strand) PQS. (**C**) Density heatmaps of G4 CUT&Tag,H3K4me3 and H3K27me3 CUT&Tag from the same cells, as well as published ATAC-seq (71), H3K4me1, H3K27ac, H3K9me3 ChIP-Seq (87). (**D**) Density heatmaps of G4, H3K4me3, H3K27me3 CUT&Tag at representative groups (~2000 genes per group) of highly-expressed, mediumly-expressed, low/non-expressed and bivalent genes. (**E**) Scatterplot showing the correlation of G4 CUT&Tag signal and CpG content at TSS regions. (**F**) Scatterplot showing the anti-correlation of G4 CUT&Tag and CpG methylation (89) at TSS regions. (**G**) Density heatmaps of G4 CUT&Tag, CpG content and CpG methylation at G4 peaks. (**H**) Density heatmaps of G4 CUT&Tag, CpG content and CpG methylation at TSS regions. (**I**) Density heatmaps of G4 qCUT&Tag in ESC and NPC, as well as H3K4me1 and H3K27ac, at active enhancer (active), primed enhancer (primed) and poised enhancer (poised) regions (62)

Supplementary Figure 3

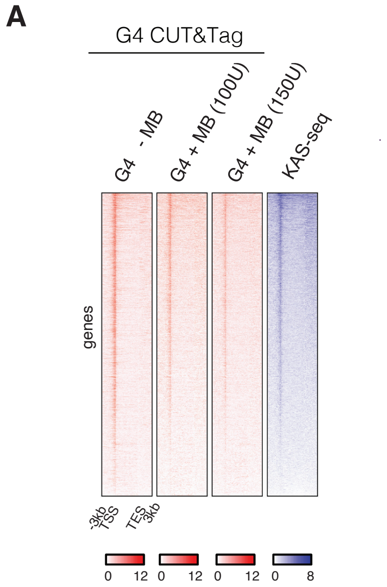

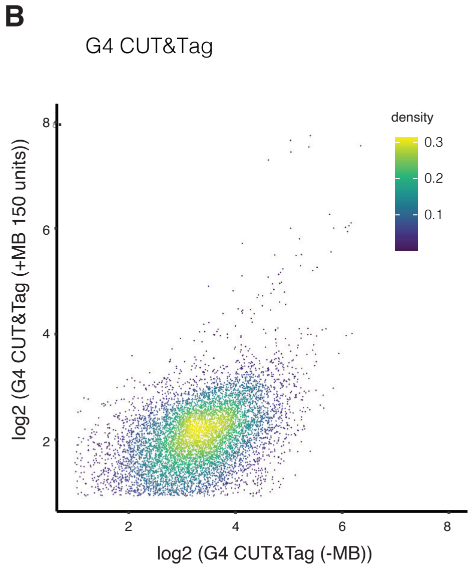

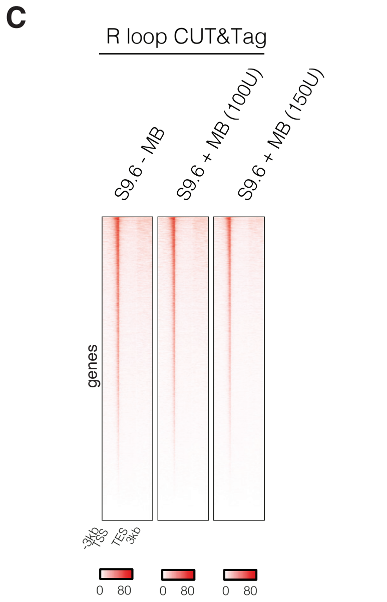

Supplementary Figure 3: Single-strand specific endonuclease treatment. (**A**) Density heatmaps of mock-treated G4 CUT&Tag, 100U MB-treated G4 CUT&Tag,150U MB-treated G4 CUT&Tag and KAS-seq (72) at gene-coding regions. (**B**) Scatterplot of G4 peaks showing the relationship of native G4 CUT&Tag and 150U MB-treated G4 CUT&Tag. (**C**) Density heatmaps of mock-treated R-loop qCUT&Tag, 100U MB-treated R-loop qCUT&Tag, 150U MB-treated R-loop qCUT&Tag at gene-coding regions.

Supplementary Figure 4

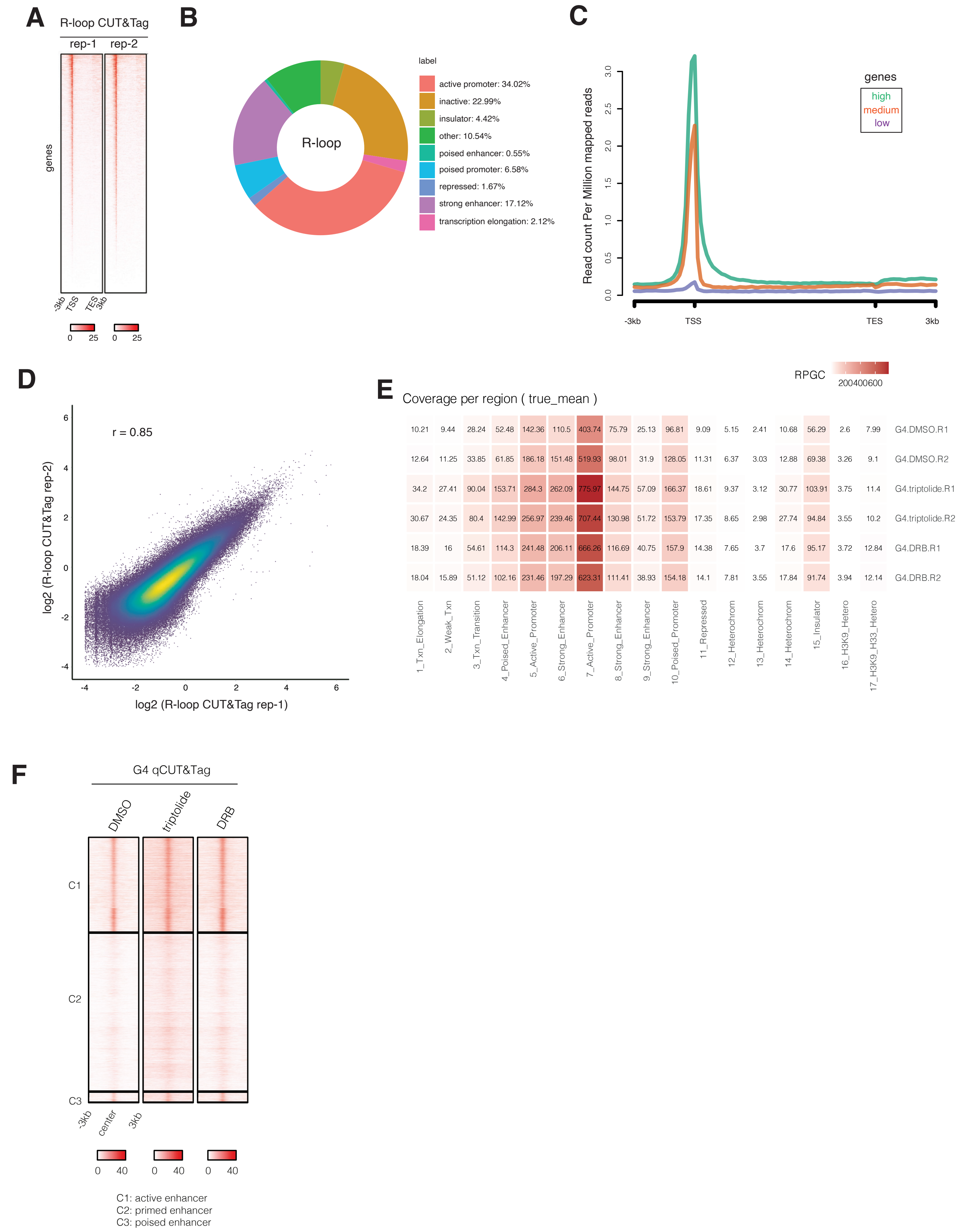

Supplementary Figure 4: Genome-wide coincidence of R-loops and G4s. (**A**) Density heatmaps showing the reproducibility of R-loop CUT&Tag replicates at gene-coding regions. (**B**) Annotation of high-confidence R-loop peaks with different functional genomic features as defined by ChromHMM (70). (**C**) Average R-loop CUT&Tag signals at high-expressed, medium-expressed and low/non-expressed gene-coding regions. Gene body and 3kb upstream of TSS and 3kb downstream of TES is shown. (**D**) Scatterplot showing the reproducibility of R-loop CUT&Tag replicates. Pearson correlation was calculated. (**E**) Heatmap showing G4 qCUT&Tag signal over ChromHMM regions in ESC with DMSO, DRB and triptolide treatment. (**F**) G4 qCUT&Tag signal over enhancers in ESC with DMSO, DRB and triptolide treatment.

Supplementary Figure 5

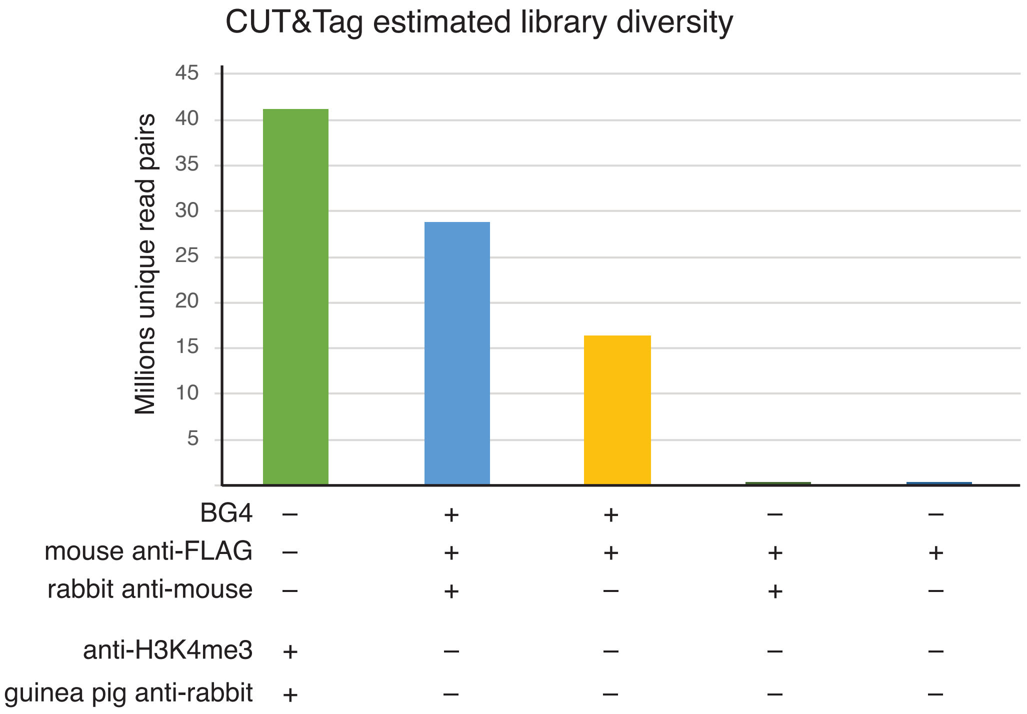

Supplementary Figure 5: Additional G4 CUT&Tag controls. Boxplot showing the estimated library diversity from picard MarkDuplicate tool of G4 CUT&Tag experiment and controls as indicated.

Supplementary Figure 6

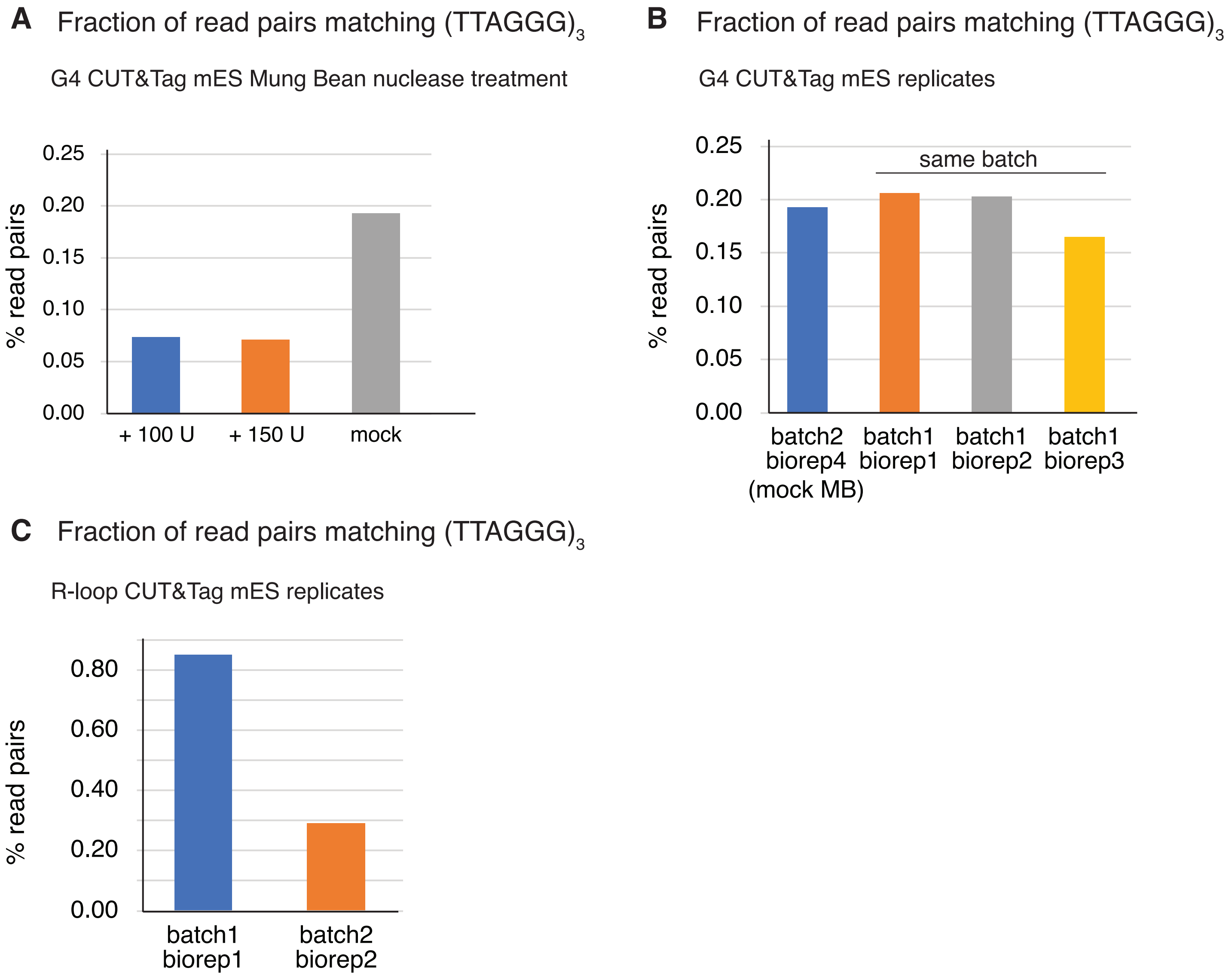

Supplementary Figure 6: Quantification of telomeric sequences in CUT&Tag data
(**A**) Boxplot showing the fraction of read pairs in G4 CUT&Tag libraries matching a telomeric sequence search $(TTAGGG)_3$ in the Mung Bean nuclease treatment experiment. (**B**) Boxplot showing the fraction of read pairs in G4 CUT&Tag libraries matching a telomeric sequence search $(TTAGGG)_3$ in four untreated, wildtype ESC replicates (biological replicates, processed in two batches). (**C**) Boxplot showing the fraction of read pairs in R-loop CUT&Tag libraries matching a telomeric sequence search $(TTAGGG)_3$
